# Factors associated with the achievement of biological disease-modifying antirheumatic drug-free remission in rheumatoid arthritis: the ANSWER cohort study

**DOI:** 10.1186/s13075-018-1673-1

**Published:** 2018-08-03

**Authors:** Motomu Hashimoto, Moritoshi Furu, Wararu Yamamoto, Takanori Fujimura, Ryota Hara, Masaki Katayama, Akira Ohnishi, Kengo Akashi, Shuzo Yoshida, Koji Nagai, Yonsu Son, Hideki Amuro, Toru Hirano, Kosuke Ebina, Ryuji Uozumi, Hiromu Ito, Masao Tanaka, Koichiro Ohmura, Takao Fujii, Tsuneyo Mimori

**Affiliations:** 1 Department of Advanced Medicine for Rheumatic Diseases, Graduate School of Medicine, Kyoto University, 53 Kawahara-cho, Shogoin, Sakyo-ku, Kyoto, 606-8507 Japan; 2 Department of Orthopedic Surgery, Graduate School of Medicine, Kyoto University, Kyoto, Japan; 3 Department of Health Information Management, Kurashiki Sweet Hospital, Kurashiki, Japan; 4 The Center for Rheumatic Diseases, Nara Medical University, Nara, Japan; 5 Department of Rheumatology, Osaka Red Cross Hospital, Osaka, Japan; 6 Department of Rheumatology and Clinical Immunology, Kobe University Graduate School of Medicine, Kobe, Japan; 7 Department of Internal Medicine (IV), Osaka Medical College, Osaka, Japan; 8 First Department of Internal Medicine, Kansai Medical University, Osaka, Japan; 9 Department of Respiratory Medicine, Allergy and Rheumatic Disease, Graduate School of Medicine, Osaka University, Osaka, Japan; 10 Department of Orthopaedic Surgery, Osaka University, Graduate School of Medicine, Osaka, Japan; 11 Department of Biomedical Statistics and Bioinformatics, Graduate School of Medicine, Kyoto University, Kyoto, Japan; 12 Department of Rheumatology and Clinical Immunology, Graduate School of Medicine, Kyoto University, Kyoto, Japan; 13 Department of Rheumatology and Clinical Immunology, Wakayama Medical University, Wakayama, Japan

**Keywords:** Rheumatoid arthritis, Biological disease-modifying antirheumatic drugs, Discontinuation, Tumor necrosis factor

## Abstract

**Background:**

Clinical remission can be maintained after the discontinuation of biological disease-modifying antirheumatic drugs (bDMARDs) in some patients with rheumatoid arthritis (RA) (bDMARD-free remission (BFR)). It is unknown which bDMARD is advantageous for achieving BFR or under which conditions BFR can be considered. This study aimed to determine the factors associated with BFR achievement in clinical practice.

**Methods:**

Patients with RA were enrolled from a Japanese multicenter observational registry. Patients with RA who achieved clinical remission (Disease Activity Score 28—C-reactive protein < 2.3) at the time of bDMARD discontinuation were included. Serial disease activities and treatment changes were followed up. BFR was considered to have failed if the disease activity exceeded the remission cutoff value or if bDMARDs were restarted.

**Results:**

Overall, 181 RA patients were included. BFR was maintained in 21.5% of patients at 1 year after bDMARD discontinuation. BFR was more successfully achieved after discontinuation of anti-tumor necrosis factor (TNF) monoclonal antibodies (TNFi(mAb)) (infliximab, adalimumab, and golimumab), followed by CTLA4-Ig (abatacept), soluble TNF receptor or Fab fragments against TNF fused with polyethylene glycol (etanercept and certolizumab), and anti-interleukin-6 receptor Ab (tocilizumab). After multivariate analysis, sustained remission (> 6 months), Boolean remission, no glucocorticoid use at the time of bDMARD discontinuation, and use of TNFi(mAb) or CTLA4-Ig remained as independent factors associated with BFR.

**Conclusions:**

BFR can be achieved in some patients with RA after bDMARD discontinuation in clinical practice. Use of TNFi(mAb) or CTLA4-Ig, sustained remission, Boolean remission, and no glucocorticoid use at the time of bDMARD discontinuation are advantageous for achieving BFR.

**Electronic supplementary material:**

The online version of this article (10.1186/s13075-018-1673-1) contains supplementary material, which is available to authorized users.

## Background

Intensive treatment strategies utilizing biological disease-modifying antirheumatic drugs (bDMARDs) have revolutionized rheumatoid arthritis (RA) treatment. Remission or low disease activity is now a realistic goal for most patients. After achieving remission, it would be advantageous if remission could be maintained without using bDMARDs (bDMARDs-free remission (BFR)) because of the associated cost-effectiveness and prevention of adverse events. It would be of clinical importance to determine which bDMARD is advantageous for achieving BFR and in what conditions BFR could be successfully maintained in daily clinical practice [1].

Discontinuation of bDMARDs after remission has been attempted in previous studies, including prospective uncontrolled trials and randomized controlled trials (RCTs) [2–17]. For example, discontinuation of infliximab (IFX) was attempted in patients with established RA, and low disease activity was maintained in 43% of patients at 1 year after discontinuation in the RRR study [5]. Similarly, remission was maintained in 58% of patients with established RA at 6 months after discontinuation of adalimumab (ADA) in the HONOR study [8]. The remission maintenance rate after discontinuation of a soluble tumor necrosis factor (TNF) receptor, etanercept (ETN), was low (28%) compared to that in the ETN continuation group (50 mg/week; 59%) or the ETN reduction group (25 mg/week: 69%) at 1 year in the PRESERVE study [11, 12]. However, the ENCOURAGE study showed that 54% of patients maintained clinical remission after discontinuation of ETN [13]. Certolizumab pegol (CZP) (Fab fragments against TNF fused with polyethylene glycol) was discontinued after achieving remission in early RA patients, and 42% of patients remained in remission 1 year after discontinuation in the C-OPERA study [14]. Discontinuation of bDMARDs has also been attempted for non-TNF inhibitors. For example, abatacept (ABT) was withdrawn along with concomitant methotrexate (MTX) treatment after achieving remission, and “drug-free remission” was maintained in 15% of patients in the AVERT study [15]. “Drug-free remission” was also maintained after discontinuation of the anti-interleukin (IL)-6 receptor antibody tocilizumab (TCZ) in 9% of patients in the DREAM study [16] and 14% of patients in the ACT-RAY study [17], respectively, after 1 year.

However, the results of these clinical trials cannot be compared because each clinical trial was conducted under different conditions, with different patient backgrounds (early or established RA), study designs (prospective uncontrolled trials or RCTs), protocols (bDMARD free or drug free), and failure outcomes (remission, low disease activity, or restart of bDMARDs) [1, 18]. BFR achievability may vary depending on the type of bDMARDs, which have different modes of action (TNF inhibitors (TNFi), CTLA4-Ig (ABT), and IL-6R inhibitors (IL-6Ri)). In addition to the typical classification of bDMARDs according to target molecules, TNFi can be classified into two groups: fully functional monoclonal antibodies with an immunoglobulin Fc portion (TNFi(mAb)), such as IFX, ADA, and golimumab (GLM); and soluble TNF absorption molecules (TNFi(R/P)), such as soluble TNF receptor (ETN) or Fab fragments against TNF fused with polyethylene glycol (CZP). It is possible that TNFi(mAb) (IFX, ADA, and GLM) might be more advantageous for achieving BFR than TNFi(R/P) (ETN and CZP) because anti-TNF monoclonal antibodies have higher cytotoxic activity against transmembrane TNF-expressing cells via complement-dependent and antibody-dependent cell-mediated cytotoxicity and inhibit granulomatous inflammation [19, 20]. However, this hypothesis cannot be tested by clinical trials that use totally different protocols. Therefore, the data from these clinical trials are not sufficient for determining how and when BFR can be successfully achieved in typical clinical practice.

Observational data from registries of patients in typical clinical practice could potentially contribute to answering these questions and providing real-world data that could be applied in daily clinical practice [21]. The Kansai Consortium for Well-being of Rheumatic Disease Patients (ANSWER) cohort was an observational multicenter registry of patients with RA in the Kansai district in Japan [22]. The data of patients at six universities (Kyoto University, Osaka University, Osaka Medical University, Kansai Medical University, Kobe University, and Nara Medial University) and associated hospitals were included. From 2011 to 2016, 4461 patients with RA were registered, and 52,654 serial disease activities were available from the database.

With the aforementioned in mind, the aim of this study was to determine which bDMARD is advantageous for achieving BFR and in what conditions BFR can be successfully achieved in typical clinical practice by utilizing the data from this multicenter observational cohort.

## Methods

### Study design and participants

We retrospectively analyzed the data for the ANSWER cohort from 2011 to 2016. Patients with RA fulfilled the 2010 ACR/European League Against Rheumatism (EULAR) criteria. In this study, we included all RA patients with Disease Activity Score 28—C-reactive protein (DAS28-CRP) < 2.3 (remission) at the time of bDMARD discontinuation to those with serial disease activity and treatment records that were fully available before and after bDMARD discontinuation. We used a DAS28-CRP remission cutoff value of 2.3, which has been validated in Japanese patients [23]. The study was approved by the ethics committee of Kyoto University (approval number R0357) as well as the ethics committees of all six institutions (Osaka University, Osaka Medical College, Kansai Medical University, Kobe University, Nara Medical University, and Osaka Red Cross Hospital). The study was conducted in accordance with the Declaration of Helsinki and written informed consent was obtained from all participants.

### Treatments

In this study, the following bDMARDs were used: IFX, ADA, GLM, ETN, CZP, ABT, and TCZ. These were categorized into four groups based on their mode of action: TNFi(mAb) (IFX, ADA, and GLM); TNFi(R/P) (ETN and CZP); CTLA4-Ig (ABT); and anti-IL-6Ri antibodies (TCZ). Other bDMARDs such as rituximab or targeted synthetic DMARDs such as JAK inhibitors were not permitted for use in patients with RA in Japan during the study period. The reasons for the discontinuation of bDMARDs were remission, inefficiency, toxic adverse events, and nontoxic reasons [22]. The conventional synthetic DMARDs (csDMARDs) used in this study were MTX, sulfasalazine, bucillamine, tacrolimus, leflunomide, iguratimod, and gold compounds. The glucocorticoids used in this study were prednisolone, methylprednisolone, and betamethasone; these were converted into prednisolone-equivalent doses.

### Outcomes

BFR failure was defined if DAS28-CRP exceeded 2.3 or if bDMARDs were restarted (including previous biologics or introduction of new bDMARDs). Changes in concomitant csDMARDs (including MTX) or glucocorticoids were not regarded as failures. If the disease activity record was not available for more than 6 months, then the case was regarded as censored at the date of the last disease activity record.

### Statistical analysis

The Kaplan–Meier method was used to estimate the median time to BFR failure from bDMARD discontinuation. A Cox proportional hazard model was used to investigate the factors associated with BFR and to obtain hazard ratios (HRs) with 95% confidence intervals (CIs). The following variables were included in univariate analysis: age, sex, disease duration, type of bDMARD, disease duration, anti-cyclic citrullinated peptide (CCP) antibody and rheumatoid factor (RF) status, bDMARD status (naïve or switched), reason for bDMARD discontinuation (physician’s intentional bDMARD discontinuation due to remission induction or not), remission maintenance period before bDMARD discontinuation, achievement of Boolean remission at the time of discontinuation, use and dosage of MTX at the time of bDMARD discontinuation, and use and dosage of glucocorticoids at the time of bDMARD discontinuation. The variables included in the multivariate analysis were selected based on the results of the univariate analysis and clinical meaningfulness. Survival curves based on the Cox proportional hazard model were evaluated for patients in each category after adjustment for covariates using direct adjusted survival estimation [24]. Two-sided *p* < 0.05 was considered statistically significant. All statistical analyses were performed using SAS statistical software, version 9.4 (SAS Institute, Cary, NC, USA).

## Results

### Patient characteristics

From 2011 to 2016, bDMARDs were used for 1307 cases in the ANSWER cohort, and serial disease activity was available for 572 cases. Based on the inclusion criteria, 181 patients with disease activity under the DAS28-CRP remission cutoff value (< 2.3) at the time of bDMARD discontinuation were included in the study and serial disease activity and treatment changes were followed up after bDMARD discontinuation.

At the time of bDMARD discontinuation, the study participants were 49 years old on average and had a disease duration of 7.6 years (Table 1). The bDMARDs used in the patients were IFX (*n* = 40), ADA (*n* = 25), GLM (*n* = 26), ETN (*n* = 22), CZP (*n* = 10), ABT (*n* = 12), and TCZ (*n* = 27). In 65.2% of patients, bDMARDs were the first-ever bDMARDs used (bDMARD-naïve). In 18.8% of patients, bDMARDs were intentionally discontinued by physicians owing to remission induction. At the time of bDMARD discontinuation, 78.5% and 42.5% of patients received MTX and glucocorticoids, respectively. All patients were treated with some DMARDs after bDMARD discontinuation, and none of them achieved drug-free remission. Patients’ baseline characteristics according to the different types of bDMARDs administered are presented in Table 1.

**Table 1:** Patient demographics at the time of bDMARD discontinuation

Type of biologic	All	TNFi(mAb)	TNFi(R/P)	CTLA4-Ig	IL-6Ri	*p* value
	(*N* = 181)	(*N* = 95)	(*N* = 32)	(*N* = 17)	(*N* = 37)	
Age (years)	49.0 ± 16.7	50.8 ± 15.7	45.9 ± 17.5	49.4 ± 18.2	46.8 ± 18.3	0.42
Female sex, *n* (%)	144 (79.6)	77 (81.1)	27 (84.4)	12 (70.6)	28 (75.7)	0.62
Disease duration (years)	7.6 ± 9.2	5.3 ± 6.9	11.2 ± 11.5	8.0 ± 7.8	10.2 ± 11.3	< 0.01
Current smoking, *n* (%)	11 (11.1)	9 (17.3)	0 (0)	1 (9.1)	1 (4.2)	0.19
bDMARD-naïve, *n* (%)	118 (65.2)	76 (80.1)	18 (56.3)	11 (64.7)	18 (48.6)	< 0.01
Discontinuation due to remission, *n* (%)	34 (18.8)	25 (26.3)	4 (12.5)	3 (17.6)	2 (5.4)	0.03
Remission maintenance period (days)	130.6 ± 185.0	162.0 ± 211.0	125.3 ± 155.8	98.3 ± 140.2	69.5 ± 135.7	0.06
DAS28-CRP	1.6 ± 0.4	1.5 ± 0.39	1.7 ± 0.4	1.7 ± 0.4	1.7 ± 0.4	< 0.01
Boolean remission achieved, *n* (%)	61 (33.7)	34 (35.8)	18 (56.3)	1 (5.9)	8 (21.6)	< 0.01
MTX use, *n* (%)	142 (78.5)	70 (73.7)	29 (90.6)	10 (58.8)	33 (89.2)	0.01
MTX dose (mg/week)	7.1 ± 2.9	8.3 ± 3.0	7.5 ± 2.4	9.0 ± 3.6	7.5 ± 2.6	0.11
Glucocorticoid use, *n* (%)	77 (42.5)	40 (42.1)	8 (25.0)	12 (70.6)	17 (45.9)	0.02
Glucocorticoid dose (mg/day)	5.9 ± 9.5	7.4 ± 12.9	4.5 ± 3.6	4.9 ± 2.0	3.9 ± 2.2	0.44
ACPA positive, *n* (%)	125 (86.2)	68 (89.5)	26 (34.2)	8 (53.3)	26 (86.7)	0.54
RF positive, *n* (%)	114 (77.0)	57 (75.0)	15 (62.5)	13 (86.7)	29 (87.9)	0.04

Demographic and clinical characteristics at the time of bDMARD discontinuation summarized as means ± standard deviations for continuous data and as numbers (percentages) for categorical data. Analysis of variance and the chi-squared test were used to compare the clinical characteristics among different groups for continuous variables and categorical variables, respectively

*bDMARD* biological disease-modifying antirheumatic drug, *TNFi(mAb)* monoclonal antibodies against TNF (infliximab, adalimumab, and golimumab), *TNFi(R/P)* soluble TNF receptor or Fab fragments against TNF fused with polyethylene glycol (etanercept and certolizumab), *CTLA4-Ig* abatacept, *IL-6Ri* interleukin-6 receptor inhibitor (tocilizumab), *DAS28-CRP* Disease Activity Score 28—C-reactive protein, *MTX* methotrexate, *ACPA* anti-citrullinated protein antibodies, *RF* rheumatoid factor, *TNF* tumor necrosis factor

### Maintenance of BFR

After bDMARD discontinuation, the BFR maintenance rates were 21.5% and 12.2% at 1 and 2 years, respectively, based on the Kaplan–Meier method. The median duration until BFR failure was 70 days (range 58–93 days). BFR failed because of disease activity flares and reinitiation of bDMARDs in 61.2% and 48.8% of patients, respectively.

### Types of bDMARDs and maintenance of BFR

**Fig. 1:**
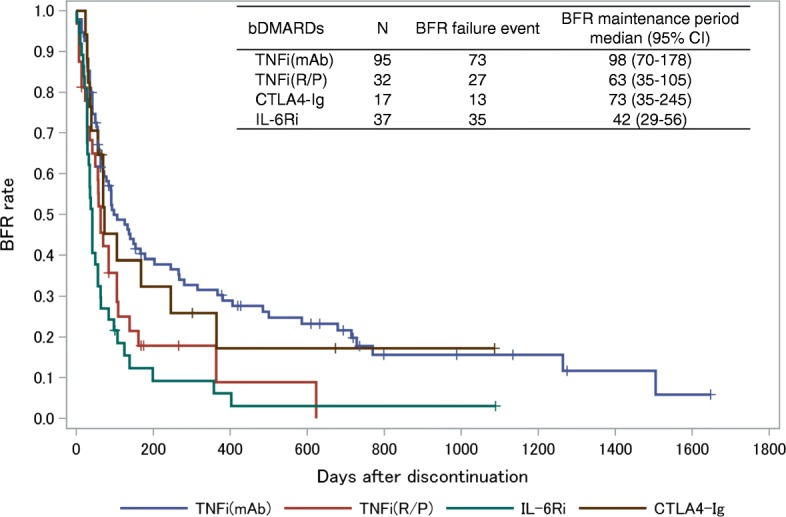
Kaplan–Meier survival curve for maintaining bDMARD-free remission after discontinuation of different types of bDMARDs. *X* axis represents days after bDMARD discontinuation. *Y* axis represents rates of maintained BFR. BFR failure defined if DAS28-CRP exceeded 2.3 or if bDMARDs restarted. If disease activity not available for more than 6 months, patient was regarded as censored case at date of last disease activity record. Kaplan–Meier method used to estimate BFR maintenance time. bDMARD biological disease-modifying antirheumatic drug, BFR biological disease-modifying antirheumatic drug-free remission, TNFi(mAb) monoclonal antibodies against TNF (infliximab, adalimumab, and golimumab), TNFi(R/P) soluble TNF receptor or Fab fragments against TNF fused with polyethylene glycol (etanercept and certolizumab), CTLA4-Ig abatacept, IL-6Ri interleukin-6 receptor inhibitor (tocilizumab), CI confidence interval, TNF tumor necrosis factor

First, we analyzed the association between the types of bDMARDs and BFR maintenance. The BFR rates according to each bDMARD are shown in Additional file 1: Figure S1. Interestingly, among multiple TNF inhibitors, the BFR rate was clearly different between patients administered TNFi(mAb) (IFX, ADA, GLM) and those administered TNFi(R/P) (ETN and CZP) (Fig. 1). The longest median BFR maintenance period was after TNFi(mAb) administration, followed by CTLA4Ig, TNFi(R/P), and IL-6Ri (Table 2). TNFi(mAb) use was associated with a decreased risk of BFR failure compared with TNFi(R/P) and IL-6Ri use (Table 2). CTLA4-Ig use was associated with a decreased risk of BFR failure compared to IL-6Ri use (Table 2).

**Table 2:** Hazard ratios for bDMARD-free remission failure (univariate analysis)

Factor	HR (95% CI)	*p* value
Type of bDMARD		
TNFi(mAb)/TNFi(R/P)	0.56 (0.35–0.87)	0.01
TNFi(mAb)/CTLA4-Ig	0.87 (0.48–1.57)	0.65
TNFi(mAb)/IL-6Ri	0.42 (0.28–0.63)	< 0.01
CTLA4-Ig/TNFi(R/P)	0.64 (0.33–1.24)	0.19
CTLA4-Ig/IL-6Ri	0.48 (0.25–0.91)	0.03
TNFi(R/P)/IL-6Ri	0.75 (0.46–1.25)	0.27
Age (years)	1.00 (0.99–1.01)	0.88
Sex, female/male	0.79 (0.53–1.20)	0.26
Disease duration, < 2 years/≥ 2 years	0.63 (0.45–0.88)	0.01
Disease duration (years)	1.03 (1.01–1.05)	< 0.01
Smoking status, current/previous or never	0.62 (0.27–1.21)	0.17
Anti-CCP antibody, positive/negative	1.47 (0.84–2.81)	0.19
Rheumatoid factor, positive/negative	1.39 (0.91–2.21)	0.13
bDMARD-naïve, naïve/switch	0.56 (0.40–0.79)	< 0.01
Reason for discontinuation, remission/other reason	0.36 (0.22–0.58)	< 0.01
Boolean remission at the time of discontinuation, achieved/not achieved	0.42 (0.29–0.60)	< 0.01
Remission maintenance period before discontinuation, > 6 months/≥ 6 months	0.33 (0.22–0.50)	< 0.01
Methotrexate usage at the time of discontinuation, yes/no	0.65 (0.45–0.96)	0.03
Methotrexate dosage at the time of discontinuation (mg/week)	0.95 (0.92–0.99)	0.02
Glucocorticoid usage at the time of discontinuation, yes/no	2.03 (1.46–2.82)	< 0.01
Glucocorticoid dosage at the time of discontinuation (mg/day)	1.01 (0.98–1.02)	0.56

Patients classified based on types of bDMARDs, disease duration, anti-CCP or RF status, smoking status, bDMARD status (naïve or switched), reasons for bDMARD discontinuation, DAS28-CRP remission maintenance period before bDMARD discontinuation, achievement of Boolean remission at time of bDMARD discontinuation, and concomitant use of MTX or glucocorticoids at time of bDMARD discontinuation. HRs with 95% CIs obtained using Cox’s proportional hazard model

*bDMARD* biological disease-modifying antirheumatic drug, *HR* hazard ratio, *CI* confidence interval, *TNFi(mAb)* monoclonal antibodies against TNF (infliximab, adalimumab, and golimumab), *TNFi(R/P)* soluble TNF receptor or Fab fragments against TNF fused with polyethylene glycol (etanercept and certolizumab), *CTLA4-Ig* abatacept, *IL-6Ri* interleukin-6 receptor inhibitor (tocilizumab), *DAS28-CRP* Disease Activity Score 28—C-reactive protein, *CCP* cyclic citrullinated peptide, *TNF* tumor necrosis factor, *RF* rheumatoid factor, *MTX* methotrexate

Because the patient backgrounds were different for those treated with different bDMARDs (Table 1**)**, the BFR rate was compared only for the bDMARD-naïve patients **(**Additional file 2: Figure S2). Similar to the results in all patients, BFR was maintained the longest after withdrawal of TNFi(mAb), followed by CTLA4-Ig, TNFi(R/P), and IL-6Ri (Additional file 2: Figure S2). TNFi(mAb) use was consistently associated with a decreased risk of BFR failure compared with TNFi(R/P) use (Additional file 3: Table S1).

### Clinical factors and achievement of BFR

We analyzed clinical factors that were associated with BFR maintenance (Table 2). Shorter disease duration (< 2 years) was associated with a decreased risk of BFR failure. Anti-CCP antibody and RF status or smoking status did not significantly affect BFR failure (Table 2). bDMARD-naïve patients were at a decreased risk for BFR failure compared to bDMARD-switched patients. bDMARD discontinuation due to remission was associated with a decreased risk of BFR failure.

If remission was continuously maintained for > 6 months before bDMARD discontinuation, patients achieved better BFR. Achievement of Boolean remission at the time of bDMARD discontinuation was also significantly associated with a decreased risk of BFR failure. The use and dosage of MTX at the time of bDMARD discontinuation were associated with a decreased risk of BFR failure, whereas glucocorticoid use at the time of discontinuation inversely increased the risk of BFR failure.

### Multivariate analysis of factors associated with BFR maintenance

**Fig. 2:**
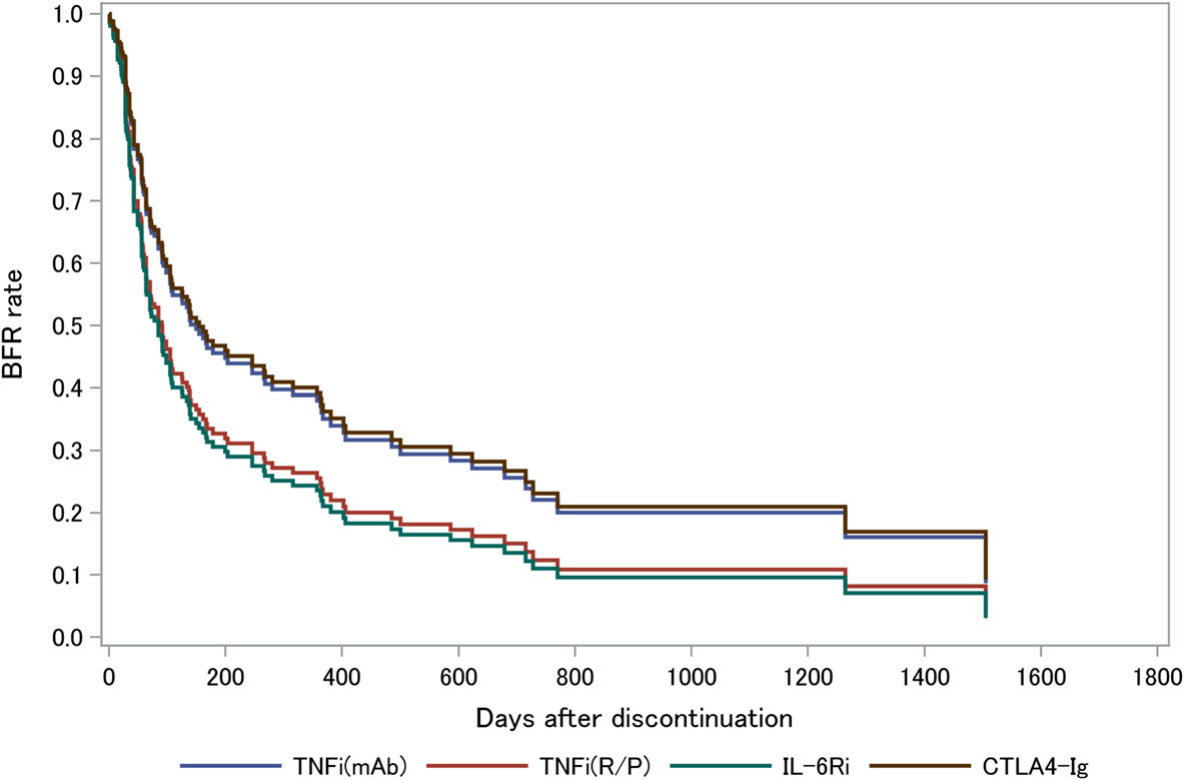
Adjusted survival curve based on Cox proportional hazard model. *X* axis represents days after bDMARD discontinuation. *Y* axis represents rates of maintained BFR. Survival curves adjusted for covariates based on Cox proportional hazard model. BFR biological disease-modifying antirheumatic drug-free remission, TNFi(mAb) monoclonal antibodies against TNF (infliximab, adalimumab, and golimumab), TNFi(R/P) soluble TNF receptor or Fab fragments against TNF fused with polyethylene glycol (etanercept and certolizumab), CTLA4-Ig abatacept, IL-6Ri interleukin-6 receptor inhibitor (tocilizumab), TNF tumor necrosis factor

We performed a multivariate analysis of factors associated with BFR failure, including the types of bDMARDs and clinical factors selected based on the results of univariate analysis and clinical significance (Table 3). In addition, adjusted survival curves for different types of bDMARDs were constructed from the results of the multivariable Cox regression model (Fig. 2). After multivariate analysis, sustained remission (> 6 months) before bDMARD discontinuation, Boolean remission at the time of bDMARD discontinuation, and glucocorticoid-free medication at the time of bDMARD discontinuation remained as independent factors associated with a decreased risk of BFR failure. After adjustment, there was no significant difference in the BFR rate between pairs of bDMARDs, except for TNFi(mAb) and IL-6Ri. However, the adjusted survival curve revealed a clear difference between use of TNFi(mAb) or CTLA4-Ig and use of TNFi(R/P) or IL-6Ri (Fig. 2). Consistently, use of TNFi(mAb) or CTLA4-Ig was significantly associated with better survival for BFR compared with use of TNFi(R/P) or IL-6Ri (HR 0.64; 95% CI 0.42–0.96; *p* = 0.03), even after adjustment.

**Table 3:** Hazard ratios for bDMARD-free remission failure (multivariate analysis)

Factor	HR (95% CI)	*p* value
Type of bDMARD		
TNFi(mAb)/TNFi(R/P)	0.67 (0.42–1.08)	0.10
TNFi(mAb)/CTLA4-Ig	1.04 (0.54–1.97)	0.91
TNFi(mAb)/IL-6Ri	0.63 (0.40–0.99)	0.05
CTLA4-Ig/TNFi(R/P)	0.65 (0.33–1.29)	0.22
CTLA4-Ig/IL-6Ri	0.61 (0.31–1.18)	0.14
TNFi(R/P)/IL-6Ri	0.93 (0.56–1.56)	0.79
Disease duration < 2 years /≥ 2 years	0.97 (0.65–1.44)	0.89
bDMARD-naïve, naïve/switch	0.85 (0.57–1.26)	0.42
Reason for discontinuation, remission/other reason	0.66 (0.38–1.14)	0.13
Boolean remission at the time of discontinuation, achieved/not achieved	0.63 (0.42–0.93)	0.02
Remission maintenance period before discontinuation, > 6 months/≥ 6 months	0.50 (0.32–0.78)	0.00
Methotrexate usage at the time of discontinuation, yes/no	1.10 (0.70–1.74)	0.67
Glucocorticoid usage at the time of discontinuation, yes/no	1.50 (1.05–2.15)	0.03

Cox’s proportional hazard model used to determine factors associated with maintenance of bDMARD-free remission in multivariate analysis. Factors included in the analysis selected according to results of univariate analysis and clinical meaningfulness

*bDMARD* biological disease-modifying antirheumatic drug, *HR* hazard ratio, *CI* confidence interval, *TNFi(mAb)* monoclonal antibodies against TNF (infliximab, adalimumab, and golimumab), *TNFi(R/P)* soluble TNF receptor or Fab fragments against TNF fused with polyethylene glycol (etanercept and certolizumab), *CTLA4-Ig* abatacept, *IL-6Ri* interleukin-6 receptor inhibitor (tocilizumab), *TNF* tumor necrosis factor

## Discussion

In this study, we analyzed favorable conditions for BFR achievement after bDMARD discontinuation in typical clinical practice using a multicenter RA registry in Japan (the ANSWER cohort). We found the following: BFR was achieved in 21.5% of patients at 1 year after bDMARD discontinuation in typical clinical practice; TNFi(mAb) or CTLA4-Ig was advantageous for achieving BFR compared with TNFi(R/P) or IL-6Ri; and sustained remission, Boolean remission, and glucocorticoid-free medication at the time of bDMARD discontinuation were important factors associated with a decreased risk of BFR failure. These findings will help decision-making in daily clinical practice when considering bDMARD discontinuation.

In this study, BFR was achieved in 21.5% of patients at 1 year after bDMARD discontinuation; the rate of BFR was lower than the rates reported in previous clinical trials [1, 3–6, 14]. The low BFR achievability in this study may be because of the stricter BFR protocol used (maintaining remission at every visit) or the diverse patient backgrounds encountered in daily clinical practice (longer disease duration, fewer bDMARD-naive patients, more patients with comorbidities, etc.). The results of this study suggest that maintaining BFR after bDMARD discontinuation is more difficult in typical clinical practice than has been reported by clinical trials.

The present results showed that TNFi(mAb) is more advantageous for achieving BFR than TNFi(R/P). This is the first study to show a substantial difference between TNFi(mAb) and TNFi(R/P) with respect to the achievability of BFR in the same observational cohort. TNFi(mAb) not only binds to soluble TNF-α but also to transmembrane TNF-α, the binding of which induces outside-to-inside signaling, leading to apoptosis of the pathogenic cells bearing transmembrane TNF-α [19]. Therefore, TNFi(mAb) but not TNFi(R/P) may not only neutralize soluble TNF but also inhibit the granuloma formation of TNF-expressing cells. The latter might create favorable conditions for successful BFR achievement after TNFi(mAb) discontinuation [19]. In addition, TNF-α inhibition might expand or restore the suppressive function of regulatory T (Treg) cells that are important for the maintenance of immunological tolerance [25–27]. Transmembrane TNF-α might be involved in this process because ADA but not ETN drives regulatory T-cell expansion via TNF-receptor 2 expressed by Treg cells [28].

CTLA4-Ig provided better survival for BFR, followed by TNFi(mAb) in the unadjusted model (Fig. 1), whereas it was almost equal to TNFi(mAb) in the adjusted model (Fig. 2). Since CTLA4-Ig targets CD4 T cells upstream of the pathological condition of RA, it might be easier to maintain a good condition even after discontinuation of bDMARDs. In fact, CTLA4-Ig reduces the number of follicular helper T cells and consequently reduces the number of switched memory B cells and autoantibodies, which may favor BFR achievement [29, 30].

It is known that IL-6 inhibition increases Treg and reduces effector T cells, which can create favorable conditions for immunological tolerance [30]. However, the BFR rate after IL-6Ri use was not as high as that associated with TNFi(mAb) or CTLA4-Ig use. Since the IL-6 signal is restored after discontinuation of TCZ, it is possible that a Treg-dominant condition might be reversed after withdrawal of IL-6Ri. Alternatively, DAS28-CRP remission might not be suited as the cutoff value considering BFR after TCZ therapy because TCZ masks CRP production and DAS28-CRP remission by TCZ can be overestimated.

Even using any bDMARDs, BFR can be achieved only in 21.5% of patients at 1 year after bDMARD discontinuation. This result suggests that immunological tolerance that could lead to long-lasting BFR has not yet been established after current bDMARD therapies, and there are still unmet needs for an RA “cure.”

This study demonstrated that BFR can be successfully achieved after achieving sustained and strict remission at the time of bDMARD discontinuation (Table 3). This result is mostly consistent with previous recommendations and the consensus for bDMARD discontinuation [1]. The importance of minimal disease activity for > 6 months has been indicated in clinical trials [1]. The importance of achieving more stringent remission than DAS28 remission before withdrawing bDMARDs has been indicated by previous studies; for example, using a lower DAS28-CRP cutoff value or the absence of Doppler signals on an ultrasonogram [5, 8, 31]. This study showed that sustained and stringent remission at the time of bDMARD discontinuation is important for successfully achieving BFR, not only in clinical trials but also in real-world clinical practice.

This study also showed that no glucocorticoid use at the time of bDMARD discontinuation is important for achieving BFR. The importance of tapering the glucocorticoid dose before bDMARD discontinuation has been suggested in the EULAR recommendations, which state the following: “If a patient is in persistent remission after having tapered glucocorticoids, one can consider tapering bDMARDs” [32]. However, the clinical evidence supporting this recommendation is insufficient. The present study strongly suggests that the glucocorticoid dose should first be tapered when considering bDMARD discontinuation because the use of glucocorticoids at the time of bDMARD discontinuation was associated with failure of BFR in the real-world observational cohort (Table 3).

The present study has several limitations. First, the number of patients was small. Even including a multicenter cohort, serial disease activity at every visit was available only in limited cases. Therefore, the present results need to be confirmed by future studies including a larger number of participants. Second, due to the small number of study participants, all patients who met the inclusion and exclusion criteria were included regardless of the reasons for drug discontinuation. The reason for drug discontinuation may have affected the BFR survival time, although we adjusted for discontinuation due to remission in multivariate analysis. Third, since this study had a retrospective design and used an observational cohort from daily clinical practice, the unknown background factors (e.g., the use of csDMARDs other than MTX or disease status at the initiation of bDMARDs) may have affected the results. Finally, the radiographic progression of joint destruction was not evaluated in this study. Notably, bDMARDs have strong protective activity against bone destruction; therefore, radiographic destruction can be inhibited by bDMARD use, even though disease activity cannot be fully controlled [33, 34]. Future studies should address whether radiographic remission can be maintained if strict BFR is maintained after bDMARD discontinuation.

## Conclusions

This study investigated the real-world conditions affecting BFR achievement in patients with RA. Although BFR is difficult to achieve in typical clinical practice, after strained and strict remission without glucocorticoid use, bDMARDs can be successfully withdrawn while retaining remission after discontinuation. Furthermore, TNFi(mAb) or CTLA4-Ig may be more advantageous for achieving BFR than TNFi(R/P) or IL-6Ri.

## Additional files

Additional file 1: **Figure S1.** Kaplan–Meier survival curve for maintaining bDMARD-free remission after discontinuation of each bDMARD. *X* axis represents days after bDMARD discontinuation. *Y* axis represents rates of maintained BFR. bDMARD biological disease-modifying anti-rheumatic drug, BFR biological disease-modifying anti-rheumatic drug-free remission, ABT abatacept, ADA adalimumab, CZP certolizumab, ETN etanercept, GLM golimumab, IFX infliximab, TCZ tocilizumab (PDF 80 kb) (PDF 79 kb)

Additional file 2: **Figure S2.** Kaplan–Meier survival curve for maintaining bDMARD-free remission after discontinuation of different types of bDMARDs in bDMARD-naïve patients. bDMARD-naïve patients classified into four groups based on types of bDMARDs. Kaplan–Meier method used to estimate BFR maintenance time. bDMARD biological disease-modifying anti-rheumatic drug, BFR biological disease-modifying anti-rheumatic drug-free remission, TNFi(mAb) monoclonal antibodies against TNF (infliximab, adalimumab, and golimumab), TNFi(R/P) soluble TNF receptor or Fab fragments against TNF fused with polyethylene glycol (etanercept and certolizumab), CTLA4-Ig abatacept, IL-6Ri interleukin-6 receptor inhibitor (tocilizumab), CI confidence interval (PDF 80 kb) (PDF 79 kb)

Additional file 3: **Table S1.** Types of bDMARDs and hazard ratios for bDMARD-free remission failure in bDMARD-naïve patients (univariate analysis). bDMARD-naïve patients classified into four groups based on types of bDMARDs. Hazard ratios with 95% CIs obtained using Cox’s proportional hazard model. CI confidence interval, bDMARD biological disease-modifying antirheumatic drug, TNFi(mAb) monoclonal antibodies against TNF (infliximab, adalimumab, and golimumab), TNFi(R/P) soluble TNF receptor or Fab fragments against TNF fused with polyethylene glycol (etanercept and certolizumab), CTLA4-Ig abatacept, IL-6Ri interleukin-6 receptor inhibitor (tocilizumab) (DOCX 15 kb) (DOCX 15 kb)

## Abbreviations

ABT: Abatacept
ADA: Adalimumab
ANSWER: Kansai Consortium for Well-being of Rheumatic Disease Patients
bDMARD: Biological disease-modifying antirheumatic drug
BFR: bDMARD-free remission
CCP: Cyclic citrullinated peptide
CI: Confidence interval
csDMARD: Conventional synthetic DMARD
CZP: Certolizumab pegol
DAS28-CRP: Disease Activity Score 28—C-reactive protein
ETN: Etanercept
EULAR: European League Against Rheumatism
GLM: Golimumab
HR: Hazard ratio
IFX: Infliximab
IL: Interleukin
MTX: Methotrexate
RA: Rheumatoid arthritis
RCT: Randomized controlled trial
RF: Rheumatoid factor
TCZ: Tocilizumab
TNFi: TNF inhibitor
TNFi(mAb): Monoclonal antibodies against TNF
TNFi(R/P): Soluble TNF receptor or Fab fragments against TNF fused with polyethylene glycol
