# Supplementary figures and images for: Factors associated with the achievement of biological disease-modifying antirheumatic drug-free remission in rheumatoid arthritis: the ANSWER cohort study
Source: Arthritis Res Ther. 2018 Aug 3;20:165. doi: 10.1186/s13075-018-1673-1 (PMC6091083; doi:10.1186/s13075-018-1673-1)

# Supplementary Figure 1

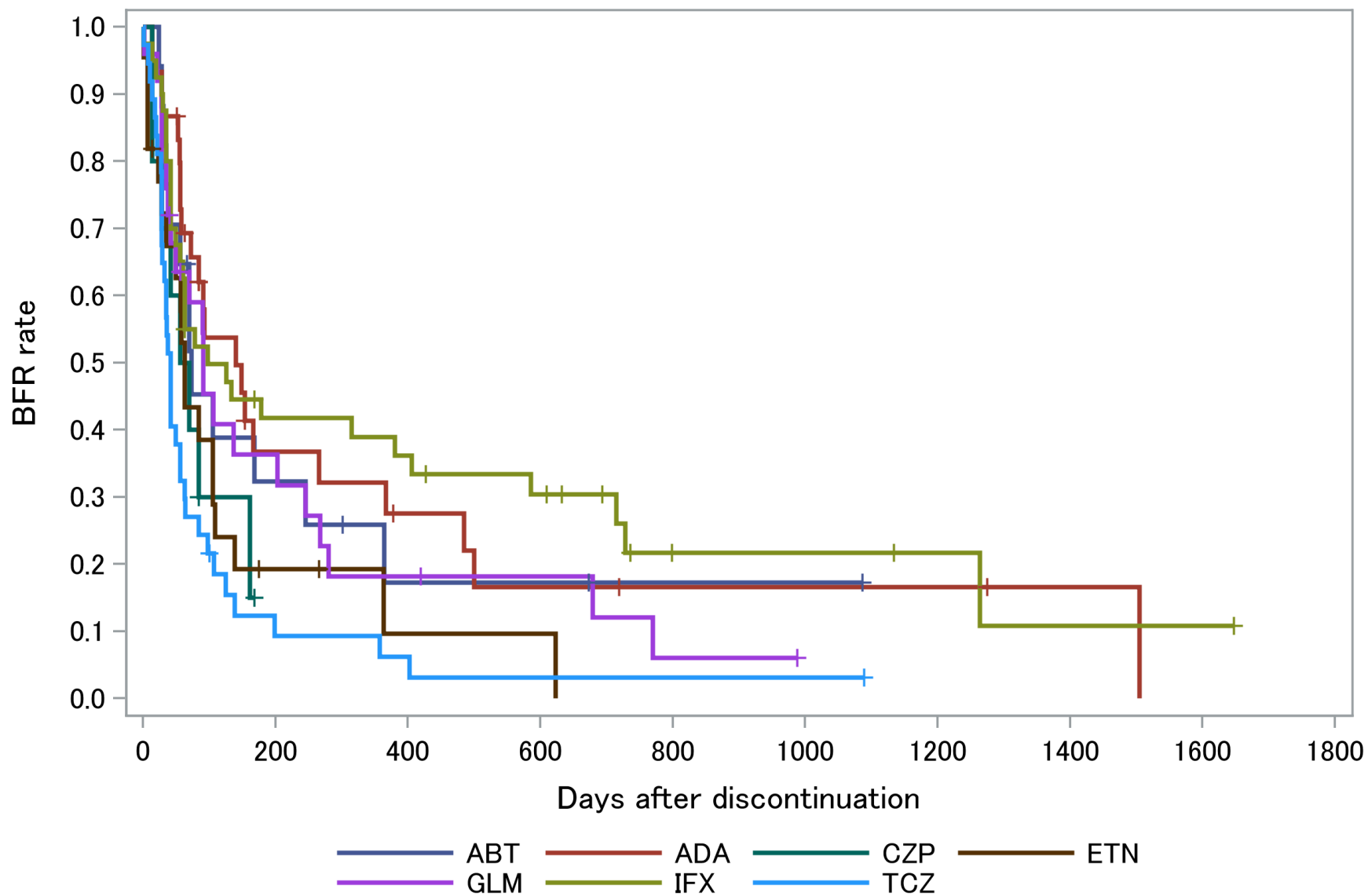

Supplement: Supplementary file 1 — Figure S1. Kaplan–Meier survival curve for maintaining bDMARD-free remission after discontinuation of each bDMARD. X axis represents days after bDMARD discontinuation. Y axis represents rates of maintained BFR. bDMARD biological disease-modifying anti-rheumatic drug, BFR biological disease-modifying anti-rheumatic drug-free remission, ABT abatacept, ADA adalimumab, CZP certolizumab, ETN etanercept, GLM golimumab, IFX infliximab, TCZ tocilizumab (PDF 80 kb) (PDF 79 kb) [file 13075_2018_1673_MOESM1_ESM.pdf]

Supplementary Figure 2

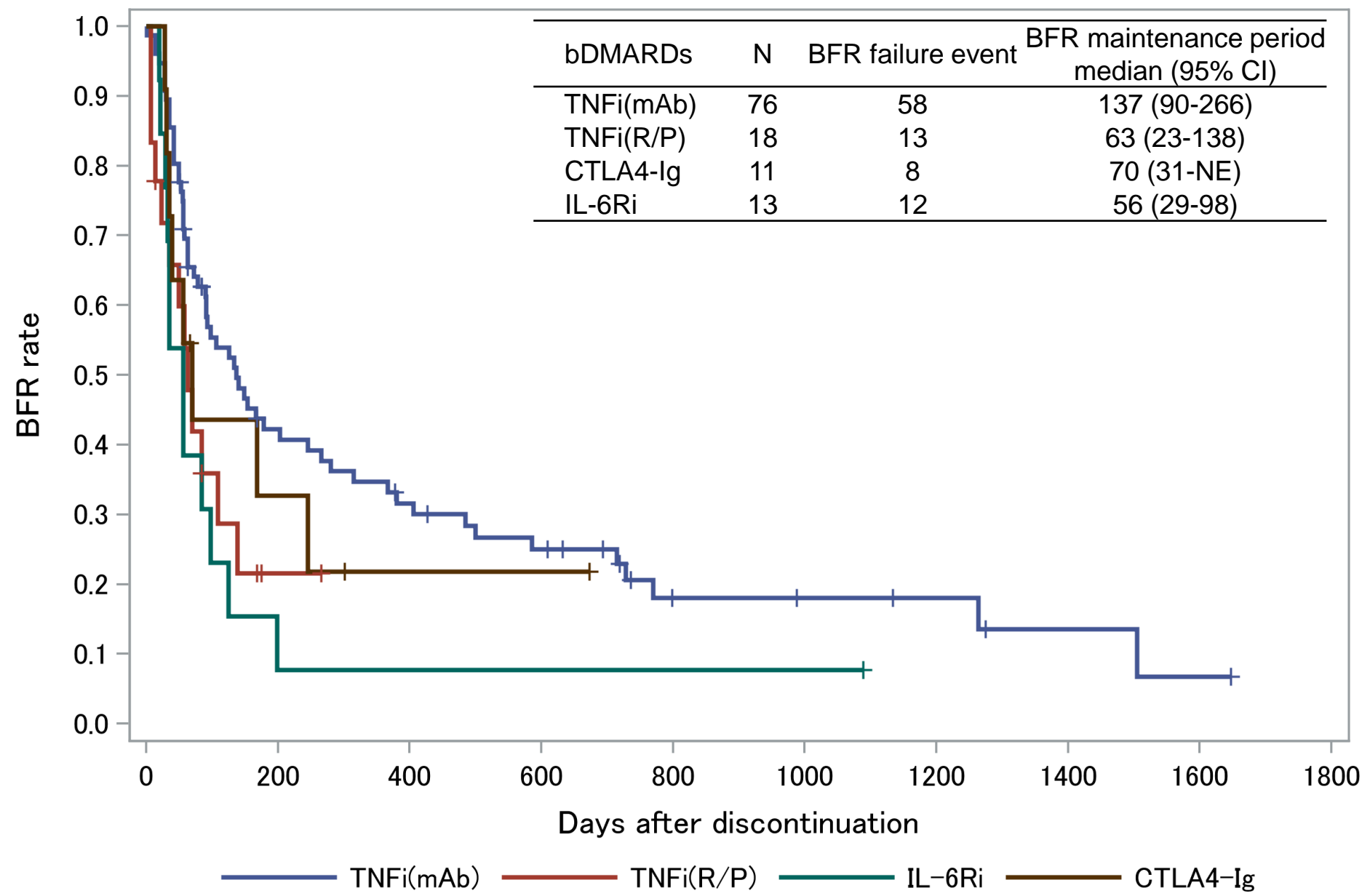

Supplement: Supplementary file 2 — Figure S2. Kaplan–Meier survival curve for maintaining bDMARD-free remission after discontinuation of different types of bDMARDs in bDMARD-naïve patients. bDMARD-naïve patients classified into four groups based on types of bDMARDs. Kaplan–Meier method used to estimate BFR maintenance time. bDMARD biological disease-modifying anti-rheumatic drug, BFR biological disease-modifying anti-rheumatic drug-free remission, TNFi(mAb) monoclonal antibodies against TNF (infliximab, adalimumab, and golimumab), TNFi(R/P) soluble TNF receptor or Fab fragments against TNF fused with polyethylene glycol (etanercept and certolizumab), CTLA4-Ig abatacept, IL-6Ri interleukin-6 receptor inhibitor (tocilizumab), CI confidence interval (PDF 80 kb) (PDF 79 kb) [file 13075_2018_1673_MOESM2_ESM.pdf]
