# Supplementary material for: Factors associated with the achievement of biological disease-modifying antirheumatic drug-free remission in rheumatoid arthritis: the ANSWER cohort study
Source: Arthritis Res Ther. 2018 Aug 3;20:165. doi: 10.1186/s13075-018-1673-1 (PMC6091083; doi:10.1186/s13075-018-1673-1)
Supplement: Supplementary file 3 — Table S1. Types of bDMARDs and hazard ratios for bDMARD-free remission failure in bDMARD-naïve patients (univariate analysis). bDMARD-naïve patients classified into four groups based on types of bDMARDs. Hazard ratios with 95% CIs obtained using Cox’s proportional hazard model. CI confidence interval, bDMARD biological disease-modifying antirheumatic drug, TNFi(mAb) monoclonal antibodies against TNF (infliximab, adalimumab, and golimumab), TNFi(R/P) soluble TNF receptor or Fab fragments against TNF fused with polyethylene glycol (etanercept and certolizumab), CTLA4-Ig abatacept, IL-6Ri interleukin-6 receptor inhibitor (tocilizumab) (DOCX 15 kb) (DOCX 15 kb) [file 13075_2018_1673_MOESM3_ESM.docx]

**Supplementary Table1. Types of bDMARDs and hazard ratios for bDMARDs-free remission failure in bDMARDs-naïve patients (univariate analysis)**

|  |  |  |
| --- | --- | --- |
| type of bDMARDs | Hazard ratio (95% CI) | P-value |
| TNFi(mAb) / TNFi(R/P) | 0.53 (0.29-0.99) | 0.05 |
| TNFi(mAb) / CTLA4-Ig | 0.75 (0.36-1.58) | 0.45 |
| TNFi(mAb) / IL-6Ri | 0.47 (0.25-0.89) | 0.02 |
| CTLA4-Ig / TNFi(R/P) | 0.71 (0.29-1.72) | 0.45 |
| CTLA4-Ig / IL-6Ri | 0.63 (0.26-1.55) | 0.31 |
| TNFi(R/P) / IL-6Ri | 0.89 (0.40-1.95) | 0.76 |
|  |  |  |
